# Vaccine effectiveness against SARS-CoV-2 transmission to household contacts during dominance of Delta variant (B.1.617.2), the Netherlands, August to September 2021

**DOI:** 10.2807/1560-7917.ES.2021.26.44.2100977

**Published:** 2021-11-04

**Authors:** Brechje de Gier, Stijn Andeweg, Jantien A Backer, RIVM COVID-19 surveillance and epidemiology team, Susan JM Hahné, Susan van den Hof, Hester E de Melker, Mirjam J Knol, RIVM COVID-19 surveillance and epidemiology team (in addition to the named authors), RIVM COVID-19 surveillance and epidemiology team (in addition to the named authors), Agnetha Hofhuis, Anne Teirlinck, Alies van Lier, Bronke Boudewijns, Miek de Dreu, Anne-Wil Valk, Femke Jongenotter, Carolien Verstraten, Gert Broekhaar, Guido Willekens, Irene Veldhuijzen, Jan Polman, Jan van de Kassteele, Jeroen Alblas, Janneke van Heereveld, Janneke Heijne, Kirsten Bulsink, Lieke Wielders, Liselotte van Asten, Liz Jenniskens, Loes Soetens, Maarten Mulder, Maarten Schipper, Marit de Lange, Naomi Smorenburg, Nienke Neppelenbroek, Patrick van den Berg, Priscila de Oliveira Bressane Lima, Rolina van Gaalen, Sara Wijburg, Shahabeh Abbas Zadeh, Siméon de Bruijn, Senna van Iersel, Sjoerd Wierenga, Susan Lanooij, Sylvia Keijser, Tara Smit, Don Klinkenberg, Pieter de Boer, Scott McDonald, Amber Maxwell, Annabel Niessen, Danytza Berry, Daphne van Wees, Dimphey van Meijeren, Eric RA Vos, Frederika Dijkstra, Jeanet Kemmeren, Kylie Ainslie, Marit Middeldorp, Marjolein Kooijman, Timor Faber, Albert Jan van Hoek, Eveline Geubbels, Birgit van Benthem, Jacco Wallinga, Rianne van Gageldonk-Lafeber

**Affiliations:** 1 Center for Infectious Disease Control, National Institute for Public Health and the Environment (RIVM), Bilthoven, the Netherlands; 2 The members of this group (in addition to the named authors) are listed under Investigators

**Keywords:** SARS-CoV-2, transmission, household study, COVID-19, vaccine effectiveness

## Abstract

We estimated SARS-CoV-2 vaccine effectiveness against onward transmission by comparing secondary attack rates among household members for vaccinated and unvaccinated index cases, based on source and contact tracing data collected when the Delta variant was dominant. Effectiveness of full vaccination of the index case against transmission to unvaccinated and fully vaccinated household contacts, respectively, was 63% (95% confidence interval (CI): 46–75) and 40% (95% CI: 20–54), in addition to the direct protection of vaccination of contacts against infection.

In early August 2021, we reported vaccine effectiveness against severe acute respiratory syndrome coronavirus 2 (SARS-CoV-2) transmission and infections among household and other close contacts of confirmed cases in the Netherlands [1]. That study was based on source and contact tracing data collected from February to May 2021, when the wildtype and Alpha variant of SARS-CoV-2 (Phylogenetic Assignment of Named Global Outbreak (Pango) lineage designation B.1.1.7) were dominating. From 29 May to 4 July 2021, the Delta variant (B.1.617.2) took over and became dominant, with over 85% Delta variant among sequenced isolates starting from 5 July.

## Source and contact tracing

A large increase in notified coronavirus disease (COVID-19) cases at the end of June 2021 resulted in a shortage of source and contact tracing capacity at the Municipal Health Services (MHS) in July and the beginning of August (Figure). Therefore, our analysis of vaccine effectiveness against transmission (VET) of the Delta variant was only possible for data collected after full source and contact tracing was resumed on 9 August 2021. We ended our study period on 24 September 2021 because since 25 September, unvaccinated people have been required to present a negative test or proof of recovery to enter bars, restaurants and events, which will impact testing behaviour differentially by vaccination status [2]. During the study period, more than 97% of sequenced Dutch isolates were identified as Delta variant [3].

**Figure:**
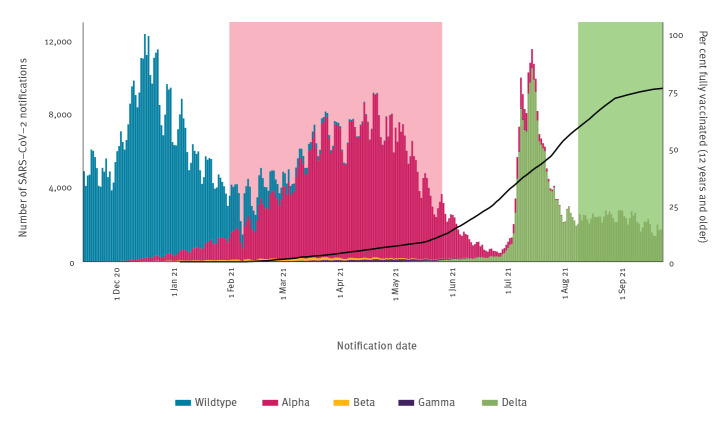
Notifications of positive SARS-CoV-2 tests per day by symptom onset, and percentage of the population fully vaccinated^a^, the Netherlands, 13 November 2020–24 September 2021 (n = 1,554,825)

SARS-CoV-2: severe acute respiratory syndrome coronavirus 2.

^a^ Full vaccination defined as 14 or more days after the second-dose of Comirnaty (BNT162b2 mRNA; BioNTech-Pfizer, Mainz, Germany/New York, United States), Spikevax (mRNA-1273, Moderna, Cambridge, United States) or Vaxzevria (ChAdOx1 nCoV-19;  Oxford-AstraZeneca, Cambridge, United Kingdom) or 28 or more days after one-dose of Janssen COVID-19 vaccine (Ad26.COV2-S, Janssen-Cilag International NV, Beerse, Belgium).

Light green area: study period presented here; pink area: study period of our previous report [1]. The share of SARS-CoV-2 variant results from fitting exponential growth curves to weekly surveillance data of sequenced samples [3].

A case was defined as a person with a positive SARS-CoV-2 PCR, loop mediated isothermal amplification (LAMP) or antigen test. Until July 2021, all household contacts of confirmed cases had to quarantine for 10 days, and were urged to get tested on Day 1 and Day 5 after exposure or in case of symptoms. If a contact was tested negative on Day 5, they could end the quarantine. On 8 July 2021, a policy change was implemented, and fully vaccinated household contacts of confirmed cases no longer had to quarantine. These fully vaccinated contacts were still strongly advised to get tested on Day 5 and to practice physical distancing until Day 10.

A full description of the data and methods used can be found in our previous report [1]. In short, a pseudonymised minimal contact monitoring dataset was used, with additional data on index cases (including vaccination status and symptom onset date) extracted from the national infectious disease notification registry. The VET was estimated by comparing the secondary attack rate (SAR) among household contacts of confirmed index cases by vaccination status of the index case: 1 − (SAR _vaccinated index_/SAR _unvaccinated index_) × 100%. 

An index case was a person with a positive SARS-CoV-2 test who, according to the source tracing interview, was most probably not infected at home. Index cases and household contacts 12 years or older were included in the analysis, as all residents in the Netherlands older than 12 years have been eligible for vaccination since July 2021. 

Partly vaccinated individuals were defined as those who had received the first dose of a two-dose schedule with a time since vaccination of at least 14 days. Fully vaccinated was defined as having completed a two-dose schedule with a time since vaccination of at least 14 days, or a one-dose schedule with a time since vaccination of at least 28 days, consistent with the definitions of ‘fully vaccinated’ in the Dutch vaccination certificate.

## Vaccine effectiveness against transmission

We estimated the VET using a binomial generalised linear model. For parameter fitting we used the generalised estimating equations approach with exchangeable correlation structure to account for clustering of contacts belonging to the same index case, using package geepack in R version 4.0.5 (R Foundation, Vienna, Austria) [4,5]. All models included age group of the index and contact (12–17, 18–29, 30–49, 50–74 and ≥ 75 years) and week of notification date of the index case as covariates. We stratified the analyses by vaccination status of the contacts.

The final dataset contained 7,771 contacts of 4,921 index cases. Of the contacts, 4,189 (53.9%) were fully vaccinated and 2,941 were unvaccinated (37.8%). Of the index cases, 2,641 (53.7%) were unvaccinated and 1,740 (35.4%) were fully vaccinated, which is a coverage much lower than in the general population (71% among adults at the start of the study period) reflecting a protective effect of the COVID-19 vaccination against infection [6]. Characteristics of index cases and contacts are shown in Table 1. Vaccination status by age reflects the roll-out of vaccination from old to young. Table 2 shows the vaccination status of contacts by vaccination status of index cases. For the unvaccinated index cases, 59.1% of household contacts were unvaccinated as well, while only 11.6% of household contacts of vaccinated index cases were unvaccinated.

**Table 1:** Characteristics of SARS-CoV-2 index cases, by vaccination status of the index and characteristics of contacts, by vaccination status of the contact, the Netherlands, August–September 2021 (n = 4,921 index cases, n= 7,771 contacts)

Characteristics	Index cases						Household contacts					
	Unvaccinated		Partly vaccinated		Fully vaccinated		Unvaccinated		Partly vaccinated		Fully vaccinated	
Total	2,641		540		1,740		2,941		641		4,189	
	n	%	n	%	n	%	n	%	n	%	n	%
Gender												
Female	1,480	56	280	52	871	50	1,517	52	313	49	2,106	50
Male	1,161	44	260	48	869	50	1,379	47	320	50	2,026	48
Unknown/other	0	0	0	0	0	0	45	2	8	1	57	1
Age group (years)												
12–17	1,005	38	174	32	45	3	903	31	172	27	127	3
18–29	823	31	229	42	549	32	718	24	216	34	673	16
30–49	616	23	101	19	438	25	910	31	176	27	1,460	35
50–74	183	7	33	6	631	36	383	13	77	12	1,841	44
≥ 75	14	1	3	1	77	4	27	1	0	0	88	2
Vaccine received												
Comirnaty^a^	NA	NA	483	89	963	55	NA	NA	505	79	2,544	61
Spikevax^a^	NA	NA	43	8	87	5	NA	NA	53	8	389	9
Vaxzevria^a^	NA	NA	14	3	273	16	NA	NA	4	1	350	8
Janssen^a^	NA	NA	NA	NA	417	24	NA	NA	NA	NA	420	10
Unknown	NA	NA	0	0	0	0	NA	NA	79	12	486	12
Household composition												
Two adults without children	825	31	165	31	977	56	634	22	160	25	1,173	28
Two adults with child(ren)	723	27	153	28	273	16	900	31	184	29	1,041	25
Single adult with child(ren)	573	22	78	14	83	5	496	17	79	12	309	7
Other	520	20	144	27	407	23	911	31	218	34	1,666	40
Month of notification of index case												
August	1,410	53	429	79	916	53	1,542	52	486	76	2,178	52
September	1,231	47	111	21	824	47	1,399	48	155	24	2,011	48

SARS-CoV-2: severe acute respiratory syndrome coronavirus 2; NA: not applicable.

^a^ Comirnaty (BNT162b2 mRNA, BioNTech-Pfizer, Mainz, Germany/New York, United States); Spikevax (mRNA-1273, Moderna, Cambridge, United States); Vaxzevria (ChAdOx1 nCoV-19, Oxford-AstraZeneca, Cambridge, United Kingdom); COVID-19 Vaccine Janssen (Ad26.COV2-S, Janssen-Cilag International NV, Beerse, Belgium).

Percentages shown are column percentages.

**Table 2:** Vaccination status of contacts relative to vaccination status of SARS-CoV-2 index cases, the Netherlands, August–September 2021 (n = 7,771 contacts)

Vaccination status contact	Unvaccinated index (n = 4,257)		Partly vaccinated index (n = 912)		Fully vaccinated index (n = 2,602)	
	n	%	n	%	n	%
Unvaccinated	2,517	59	121	13	303	12
Partly vaccinated	235	6	177	19	229	9
Fully vaccinated	1,505	35	614	67	2,070	80

SARS-CoV-2: severe acute respiratory syndrome coronavirus 2.

Percentages shown are column percentages.

Table 3 shows a lower crude SAR among unvaccinated household contacts for vaccinated index cases compared with unvaccinated index cases (13% vs 22%) and a corresponding adjusted VET of 63% (95% confidence interval (CI): 46–75). Among fully vaccinated household contacts, the crude SAR was similar for fully vaccinated index cases and unvaccinated index cases (11% vs 12%), but this was confounded by age of the index – both SAR and proportion of vaccinated index cases were higher in the oldest age groups (Supplementary Table S1). After adjustment, the VET of full vaccination of the index case was 40% (95% CI: 20–54). 

**Table 3:** Secondary attack rate of SARS-CoV-2 infection by vaccination status of the index case (≥ 12 years) and vaccine effectiveness against transmission, the Netherlands, August–September 2021 (n = 4,921 index cases)

Analysis	Unvaccinated index			Partly vaccinated index					Fully vaccinated index				
	SAR			SAR			Crude VET	Adjusted VET^a^	SAR			Crude VET	Adjusted VET^a^
	Positive	**Total**	**%**	**Positive**	**Total**	**%**	% (95% CI)	% (95% CI)	Positive	Total	%	% (95% CI)	% (95% CI)
Unvaccinated household contacts	547	2,517	22	21	121	17	28 (−18 to 56)	38 (−2 to 62)	38	303	13	50 (28 to 65)	63 (46 to 75)
Fully vaccinated household contacts	164	1,505	11	37	614	6	46 (22 to 63)	46 (20 to 63)	256	2,070	12	−16 (−44 to 6)	40 (20 to 54)

CI: confidence interval; SAR: secondary attack rate; SARS-CoV-2: severe acute respiratory syndrome coronavirus 2, VET: vaccine effectiveness against transmission.

^a^ Adjusted for age group of the index case and contact and for week of notification of the index case.

## Discussion

We had previously found a higher VET to unvaccinated household contacts while the SARS-CoV-2 Alpha variant was predominantly circulating (73%; 95% CI: 65–79) [1]. The secondary attack rate among unvaccinated contacts was also higher during that period (31%) compared with this study dominated by circulation of the Delta variant (22%). This may be a result of increased prevalence of infection-induced immunity. In the beginning of August, around 20% of Dutch blood donors had infection-induced immunity [7]. A larger share of index cases were of a younger age (< 30 years) compared with our previous analysis, and SAR were lower for younger index cases (Supplementary Table S1). 

Our data do not contain information about negative tests. Therefore it is uncertain whether contacts tested negative or did not test at all. Even though both vaccinated and unvaccinated household contacts are advised to test on Day 5 and in case of symptoms, we cannot exclude the possibility that testing rates among household contacts became lower compared with our earlier study, leading to an underestimation of the SAR. Differences in testing behaviour between contacts of vaccinated and unvaccinated index cases could bias our VET estimates. 

During the study period, most Dutch adults had had the opportunity to receive vaccination, the coverage for 12–17-year-olds was still increasing during this period (around 60% at the end of the study period). The current vaccinated and unvaccinated populations are likely to be different in multiple aspects, such as risk behaviour, willingness to test and adherence to quarantine. These aspects might bias our VET estimates in both directions: while the perceived risk of infection might be smaller in vaccinated people because of their vaccination status, the perceived risk of infection among current unvaccinated populations could also be small. A lower risk perception in both groups may have resulted in decreased testing rates. Daily testing numbers at the MHS test locations averaged around 60,000 in spring 2021, while in August and September, this averaged around 20,000, which is also likely to be influenced by the increasing use of at-home rapid antigen tests [8]. Furthermore, vaccinated and unvaccinated people were strongly clustered within households. This reduced the power of our analysis.

It is known from the literature that the Delta variant is more transmissible than the Alpha variant and more likely to cause vaccine breakthrough infections, therefore a reduced VET for Delta compared with Alpha is not unexpected [9,10]. A recent study by Eyre et al. reported reduced transmission for vaccinated index cases, with adjusted Odds ratio estimates in line with our VET estimates for both Alpha and Delta variant [11]. Eyre et al. found that VET decreased with time since vaccination of the index case. We explored whether such a decrease was also visible in our data (Supplementary Table S2). VET estimates were indeed lower when the index case reached full vaccination status 60 or more days before. However, our data do not allow detailed analysis of VET decrease owing to small numbers and strong correlation with age and time since vaccination of the household contacts. If VET indeed declines with time since vaccination, the lower VET for the Delta compared with the Alpha variant may be (partly) due to longer time since vaccination rather than to the variant itself.

## Conclusion

Our results indicate that vaccination confers protection against onward transmission of SARS-CoV-2 from vaccinated index cases, albeit somewhat less for the Delta than for the Alpha variant. The VET to unvaccinated household contacts is higher than to vaccinated household contacts, with the latter already largely protected from infection and especially from severe disease by their own vaccine-induced immunity. The difference in VET between unvaccinated and fully vaccinated household contacts might also be attributable to differences in age distribution and/or unmeasured confounding, for example by clinical vulnerability or risk behaviour, between the two populations. Possible decreasing vaccine effectiveness against infection and against onward transmission could result in increased SARS-CoV-2 circulation among populations with high vaccine coverage. As full vaccination remains highly effective in preventing severe disease, also for the Delta variant, a high vaccination coverage remains the key to control the COVID-19 pandemic [12]. 

## Supplementary Data

134000 Supplement
