# Supplementary material for: Vaccine effectiveness against SARS-CoV-2 transmission to household contacts during dominance of Delta variant (B.1.617.2), the Netherlands, August to September 2021
Source: Euro Surveill. 2021 Nov 4;26(44):2100977. doi: 10.2807/1560-7917.ES.2021.26.44.2100977 (PMC8569927; doi:10.2807/1560-7917.ES.2021.26.44.2100977)
Supplement: Supplement [file 21-00977_deGIER_Supplement.pdf]

Supplementary tables

This supplementary material is hosted by *Eurosurveillance* as supporting information alongside the article *Vaccine effectiveness against SARS-CoV-2 transmission to household contacts during dominance of Delta variant (B.1.617.2), August-September 2021, the Netherlands* on behalf of the authors who remain responsible for the accuracy and appropriateness of the content. The same standards for ethics, copyright, attributions and permissions as for the article apply. Supplements are not edited by Eurosurveillance and the journal is not responsible for the maintenance of any links or email addresses provided therein.

Table S1. Secondary attack rates (SAR) for unvaccinated and fully vaccinated index cases, by age group of the index case.

Age group index	Unvaccinated index - SAR			Fully vaccinated index - SAR		
	positive	total	percentage	positive	total	percentage
Unvaccinated household contacts						
All ages	547	2517	22	38	303	13
12-17	139	967	14	2	7	29
18-29	140	719	19	6	57	11
30-49	187	618	30	15	158	9
50-74	76	202	38	12	77	16
75+	5	11	45	3	4	75
Fully vaccinated household contacts						
All ages	164	1505	11	256	2070	12
12-17	88	851	10	2	86	2
18-29	37	483	8	46	838	5
30-49	31	135	23	45	388	12
50-74	6	33	18	141	680	21
75+	2	3	67	22	78	28

Table S2. Secondary attack rate (SAR) of SARS-CoV-2 and vaccine effectiveness against transmission (VET) adjusted for time since full vaccination of the contact (< or >= 60 days, only in analysis of fully vaccinated contacts), age group of the index case and contact and week of notification date of the index case, stratified by time since full vaccination of the index case.

Analysis	Unvaccinated index - SAR			Index fully vaccinated < 60 days ago - SAR			Index fully vaccinated < 60 days ago - adjusted VET (%) (95% CI)	Index fully vaccinated >= 60 days ago - SAR			Index fully vaccinated >= 60 days ago - adjusted VET (%)(95% CI)
	positive	total	percentage	positive	total	percentage		positive	total	percentage	
Unvaccinated household contacts	547	2517	22	24	209	11	67 (47 to 79)	14	94	15	55 (19 to 76)
Fully vaccinated household contacts	164	1505	11	99	1278	8	57 (40 to 69)	157	792	20	28 (-4 to 50)
